# A highly accurate method for forecasting the compressor geometric variable system based on the data-driven method

**DOI:** 10.1371/journal.pone.0283108

**Published:** 2023-07-27

**Authors:** Cunjiang Xia, Yuyou Zhan, Yan Tan, Yi Gou, Wenqing Wu

**Affiliations:** Civil Aviation Flight University of China, Guanghan, 618300, China; Galgotias University, INDIA

## Abstract

To make the puzzle of aero-engines complete, understanding the law of the compressor geometric variable system is a vital part. Modeling all aspects of aero-engines quickly has been a continuous area of research since the advent of artificial intelligence (AI). However, diagnosing or predicting faults is an ancient adage, and it is vital to explore key system forecast research, particularly since traditional forecasting techniques do not account for future information of non-target parameters. In this article, based on the feasibility of forecasting the compressor geometric variable system, an enhanced ConvNeXt model utilizing the Sliding Window Algorithm mechanism is proposed. And this method takes into account the future information of non-target parameters. With the novel strategy, the issue of the forecast’s error increasing with forecast length is alleviated. As a result, in a particular condition, the error we obtained only accounts for **20.07%** of that of the standard forecast approach. Additionally, it is verified that this method can be applied to various aero-engines. Finally, experiments on several aero-engine states involving the transition state and the steady state are conducted to strengthen the plausibility and credibility of our theories. It should be noted that the foundation of each experiment is data from actual flights.

## 1. Introduction

Understanding the law hidden in the complex control systems of aero-engines is a huge leap in the field of aeronautics. And the exploration of regular models through data collection is always used in the entire process of aero-engine research. Artificial intelligence (AI) has made it feasible for researchers to quickly gain new insights about aero-engines, which were previously unreachable due to the complexity of essential systems. Presently, common fault diagnosis and classification algorithms have been applied to a variety of aero-engine systems, but due to the complexity of aero-engines and the confidentiality principle, there are also still barriers for airlines or a non-OEM (Original Equipment Manufacturer) to understanding the operation law of critical systems. Especially in aero-engine maintenance and airworthiness safety research [1,2]. As a fundamental part, the compressor must deal with stall phenomena, which can result in a number of different types of fluid dynamic instabilities [3]. As a result, the compressor geometric variable system is crucial to the process of controlling the flow path. And the area of aeronautics could benefit from a detailed grasp of the fundamental operating principles and potential developments of this system.

Based on the previous background, the introduction of the compressor geometric variable system forecast will give a significant amount of support for aero-engine design optimization and airworthiness safety monitoring [4–7]. Additionally, it should be noted that the study on compressors hardly includes forecasting and modeling of important control systems and instead primarily focuses on fault detection, fault prognosis, and flow forecasting. On the other hand, a thorough examination of the compressor geometric variable system’s prognosis will have a very favorable impact on the improvement of the control system and the safety of flights. In particular, it could serve as motivation for those who are aero-engine researchers rather than original aero-engine designers. Therefore, research into a highly accurate method for forecasting the compressor geometry variable system is extremely important from a practical standpoint.

**Fig 1:**
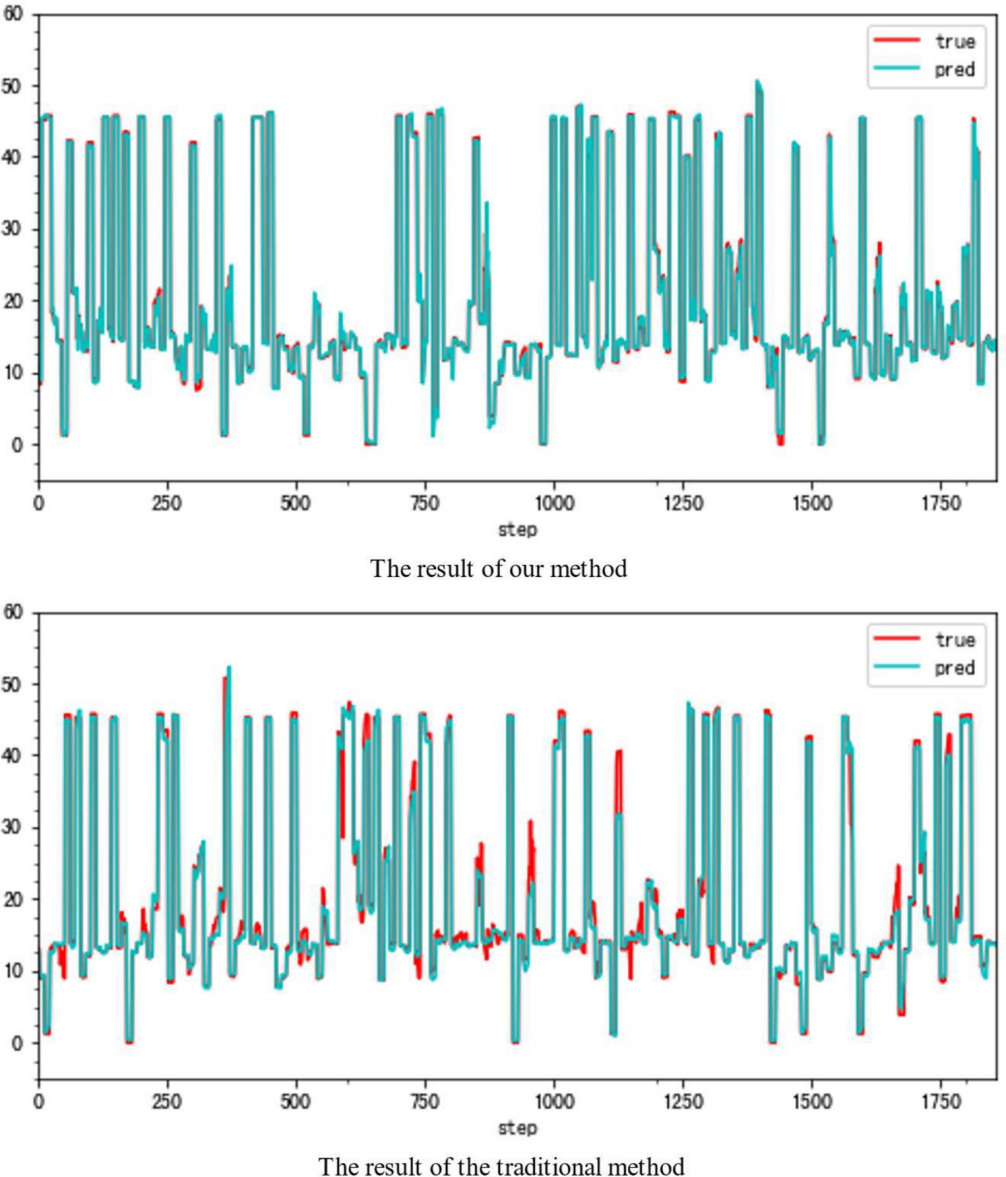
The forecasting results (VSV, Type B).

In this paper, we propose a highly accurate prediction method for the compressor geometric variable system. And Fig 1 presents parts of our results. Fig 1 shows the difference between our method and the traditional forecasting method in the same condition. The great improvement is obvious. (**79.93% fewer errors overall**)

**Fig 2:**
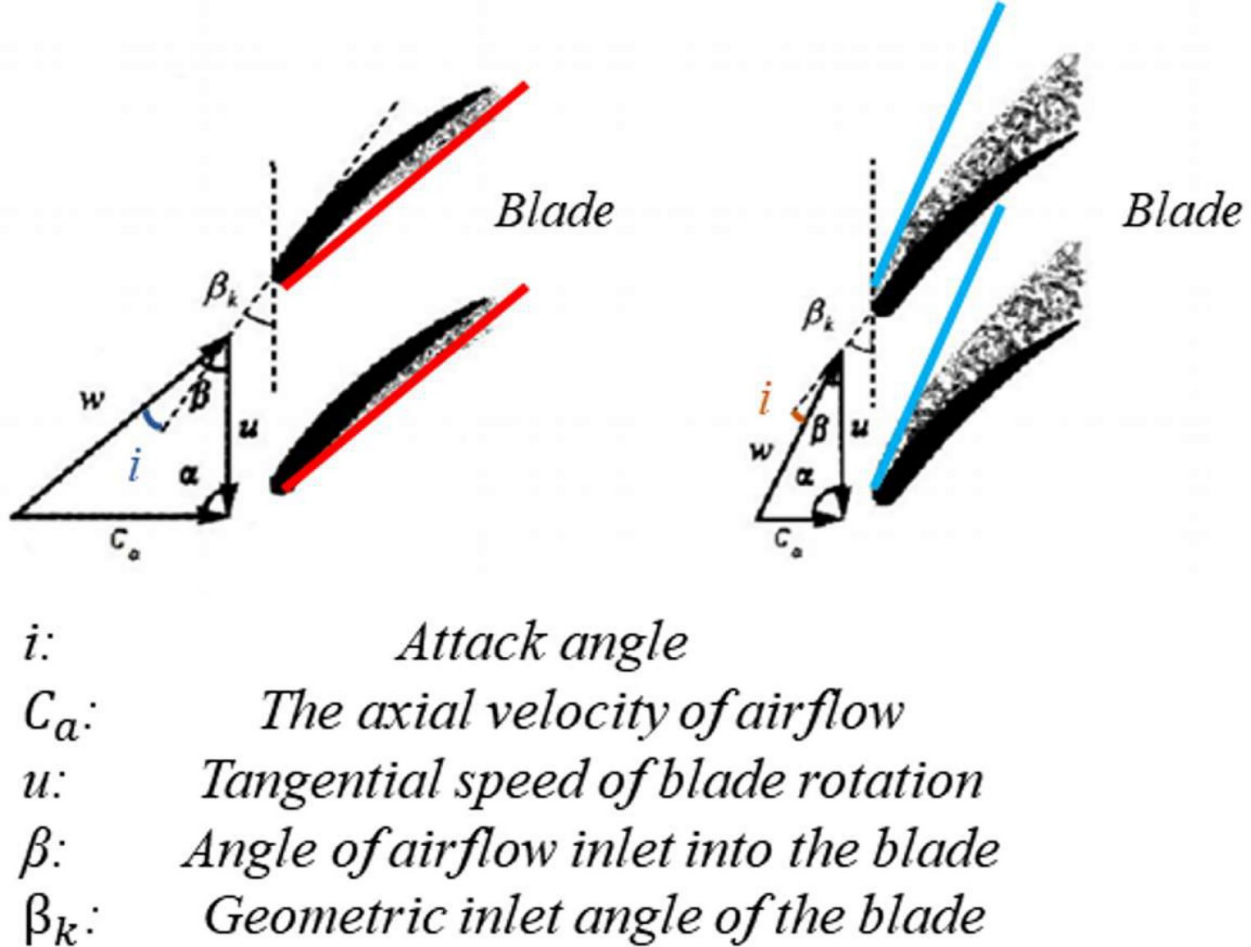
The phenomenon of burbling.

The compressor geometric variable system is a key part of the whole engine gas path system. A topic system consists of a Variable Stator Vane (VSV) system and a Variable Bleed Valve (VBV) system. In the compressor, the incoming fluid will be pressurized by first accelerating it via the kinetic energy transmitted in the rotors and then diverging channels will slow the fluid down so that the kinetic energy could be converted into potential energy [8]. However, rotating stalls and surges will make the system unstable and insecure, which means there would have a negative impact on the airworthiness of the aircraft. When the compressor speed is consistent, increasing the airflow of the compressor will increase the axial velocity of the airflow (*c*_*a*_) increase and the attack angle (*i*) will decrease or even be negative. If the negative value is too large, the separation of the blade basin occurs and a vortex zone forms in the blade basin. Although the vortex zone will not continue to expand, the separation of the blade basin will reduce the operating efficiency of the blade and the flow capacity of the airflow at the section. Hence, the results will reduce the airflow at the compressor inlet and will increase the degree of former stage attack angles. In addition, decreasing the airflow of the compressor will decrease the axial velocity of airflow (*c*_*a*_) and the attack angle (*i*) will increase. If the attack angle is too large, the airflow will separate from the back of the blade, which is known as the stall. With the compressor rotating, the stall area rotates in the same direction at a lower speed and gradually expands, which is called the rotating stall. When the rotating stall phenomenon develops further, the whole compressor path will be blocked, and even the compressor will enter the surge state. In case of the surge, the average pressure at the compressor outlet drops abruptly, the compressor blades and engine parts vibrate strongly, and even the blades break. Meanwhile, the Exhaust Gas Temperature rises, and the sound of the aero-engine changes from howling to low. And the thrust could drop suddenly, the combustion chamber could turn stop. In severe cases, the aero-engine may be damaged or even destroyed. Fig 2 shows the phenomenon of these two kinds of burbling. As a result, how accurately forecasting angles of VSV and openings of VBV systems is a meaningful and necessary research for the troubleshooting early warning and the design of aero-engine control systems.

Optimizing the angle of VSV and the opening of VBV allowed the fluid state to be corrected. And this can ensure the aero-engine works steadily and effectively [9,10].

Within a certain range, it can actually increase the system’s forecast precision by taking into account multi-parameter fusion. However, it should be emphasized that other parameters’ future tendencies should also be taken into account in addition to the target value. Because the target parameter’s forecasting error will grow due to the uncertainty of other factors. Therefore, it is a very important task worth considering how to incorporate future knowledge from other parameters into the model, which could enhance the performance of forecasts.

**Fig 3:**
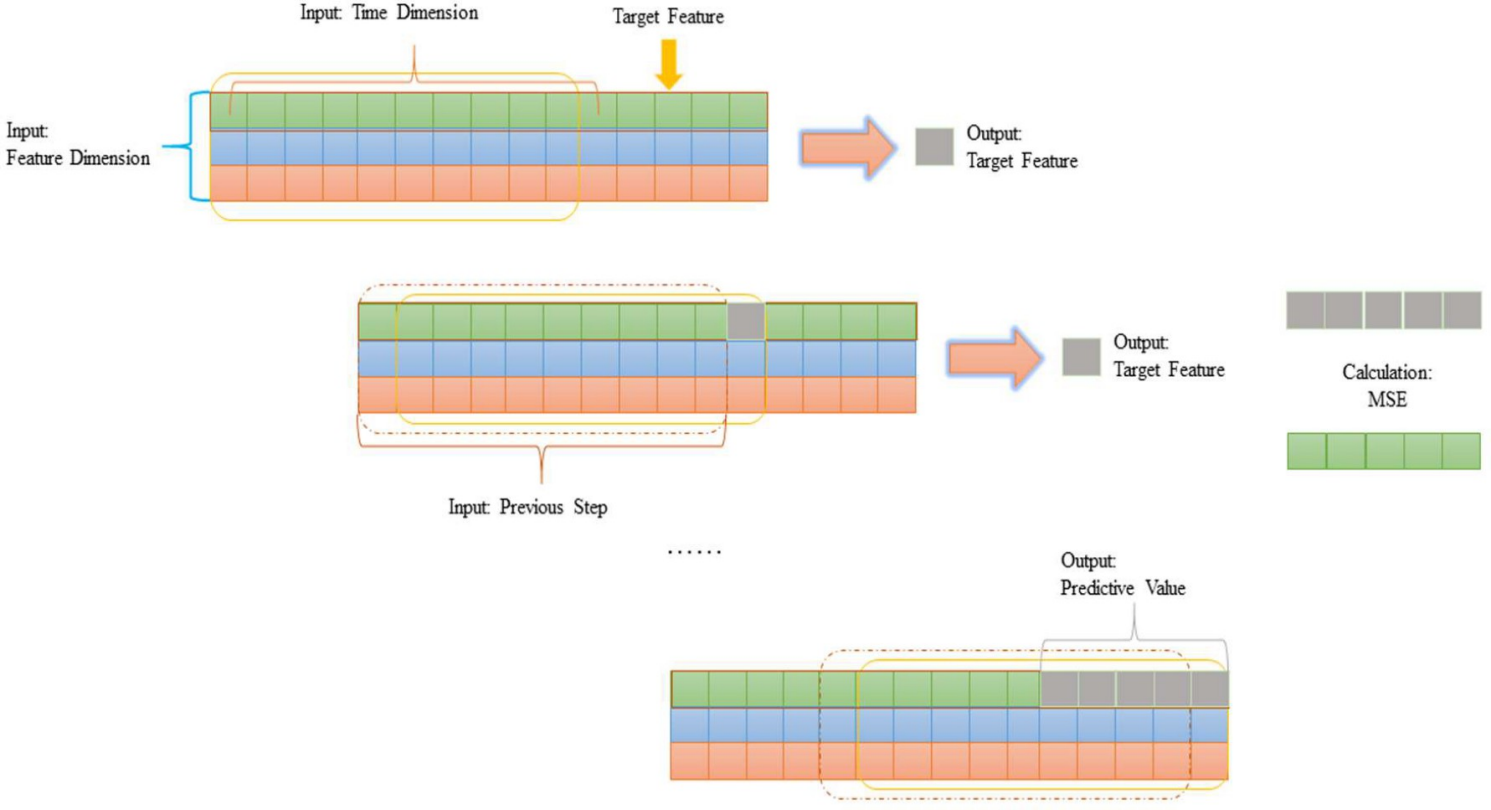
The sliding window algorithm mechanism.

To conquer this barrier, we adopt a special strategy which is called the “Sliding Window Algorithm”. The term ’Sliding Window Algorithm’ has been used to refer to situations in which the network communication in the field of computer network (Transfer Control Protocol) [11]. And our algorithm architecture is shown in Fig 3. Inspired by this technique, we designed a predictive algorithm for the compressor geometric variable system using the Sliding Window Algorithm. Our approach yields errors that are **20% to 80%** lower than those achieved using traditional techniques. To verify the effectiveness of this method, the prediction of different types of aero-engines is also tested. In addition, experiments on several aero-engine states involving the transition state and the steady state are conducted to strengthen the plausibility and credibility of our theories. Finally, the datasets used in this article are generated by aircraft acquisition systems, which means the result that the model forecasts could be more fit for actual flight situations.

## 2. Data preprocessing

### 2.1 Data source

Unlike the previous use of laboratory datasets or simulation data, the datasets we use come from aircraft data acquisition systems. Various and detailed parameters, continuous storage, easy export, and processing [12] are all advantages of our datasets. By leveraging our data, the model may learn the latent representation of key features which could represent complex operating conditions within the system. Based on the above situation and the future information of other parameters brought by the sliding window mechanism, the model could reach a high precision. These advantages make our research more credible and complete. It should be noted that the types of aero-engines we studied are dual rotor turbofan engines, hence the datasets belong to the dual rotor turbofan engine, not to others.

The operation conditions of aero-engines are divided into transition state and steady state, which involve a whole flight. Therefore, the datasets gathered by aircraft acquisition systems will belong to these two aero-engine operating states. It should be noted that datasets from both states will be randomly extracted and input into our model at the same time. Hence when the model is sufficiently complex, it could learn the patterns of these two states in training tasks and identify which state it is according to the input data during the forecasting process. As a result, the enhanced ConvNeXt model can be regarded as a general forecast model that does not need to distinguish aero-engine conditions in the data preprocessing part due to its automatic recognition capability. This method of extracting data randomly must make the transition state and steady state data samples of aero-engines be input into the model in a random way.

### 2.2 Parameter selection

In our study, forecasting the variation of VSV and VBV accurately is the main objective. Therefore, the target features are the angle of VSV (°) and the opening of VBV (°). When selecting parameters, the interaction of parameters and the technology principle of aero-engines will be involved. Furthermore, this essay makes reference to earlier works and expert technical manuals on aero-engines [13,14]. Therefore, the following parameters in Table 1 of Type A aero-engines will be chosen.

**Table 1:** Parameters Selection (A).

Parameter	Unit
Altitude	Feet
Throttle lever angle (TRL)	°
High pressure compressor inlet temperature (T25)	°C
High pressure compressor outlet temperature (T3)	°C
Total air temperature (TAT)	°C
High pressure compressor outlet pressure (PS3)	PSIA
Low pressure rotor speed (N1)	%RPM
High pressure rotor speed (N2)	%RPM
Variable stator vane (VSV)	°
Variable bleed valve (VBV)	°

Because our experiments will be tested in different types of aero-engines, as well as the aircraft data acquisition system is little different. But the selection of essential parameters of Type B aero-engines in Table 2 will do our best to remain the same.

**Table 2:** Parameters Selection (B).

Parameter	Unit
Ambient pressure (P0)	PSIA
High pressure compressor outlet pressure (PS3)	PSIA
Throttle lever angle (TRL)	°
Fan inlet temperature (T12)	°C
High pressure compressor inlet temperature (T25)	°C
Total air temperature (TAT)	°C
Low pressure rotor speed (N1)	%RPM
High pressure rotor speed (N2)	%RPM
Variable stator vane (VSV)	°
Variable bleed valve (VBV)	°

### 2.3 Time span

The term ‘step’ has been used to refer to situations in which time series data are used. In the current research, the step is defined as the frequency of data recording. It should be noted that one step can be representative of the sensor acquisition rate which is 1 Hz in this study. Correlative parameters in 100 steps will be primarily utilized in this essay to forecast 10 in the future. The reason these steps are set up as they are is that longer inputs may contain sufficient information for forecasting tasks, and short future steps that must be forecasted lead to results that are more accurate and dependable.

### 2.4 Random sampling

The method of randomly extracting a block of data is used in this work to build a powerful model. The input will randomly sample continuous 100 steps as one batch, and the output will be the VSV or VBV values in the next continuous 10 steps. By using this method, the forecasting task would be more challenging and the model might learn actual latent control features more effectively. Because the model is expected to learn the relationship of parameters changing and interacting, instead of the temporal link between flight segments [15,16]. In the experiment section, 64 batches will be randomly sampled as inputs to a round of training.

## 3. Method

In this paper, some classic and advanced learning algorithms (ResNet18, SENet18, and original ConvNeXt18) are used for comparison, because we want to explore whether this complex algorithm itself could improve the forecasting accuracy. To accomplish some goals, such as quick modeling to evaluate the theory and fitting the size of the dataset, a light and enhanced ConvNeXt model will be a very suitable helper.

### 3.1 The sliding window mechanism

The core of our method is the sliding window mechanism. In previous research, the study of forecasts is straightforward. Input data enters the model, and the model outputs expected values. However, control systems of aero-engines are very complex and multifactorial. Control object states may abruptly change as a result of the superior system’s signal and rapid changes in other parameters. The model could identify the changing pattern of the control system through input data of a certain time span (like 100 steps/HZ/second), while forecasting the future state of the controlled object is affected by the inertia of the input data. If the sudden change factor is not in the input data but coming from the future which needs to forecast, a large difference might occur between the output value and the real value. Hence an effective method should be adopted to address this defect of this traditional prediction method.

Fig 3 shows the main architecture of our model. The input is the change of the target parameter and other parameters over a period of time (100 steps or other). And the output is the predictive value of the target value in the next step. The window then slides one step forward and takes the output value just now as the value of the latest step of the target parameter. As shown in the gray grid in Fig 3. After the steps that we want to forecast, the Mean Square Error (MSE) between the predictive and the true will be calculated. Therefore, future information (latently sudden change) on other impact factors will be considered based on this series of operations when the model is computing. Based on this idea, erroneous information can be corrected during forecasting. Forecasting accuracy will also be significantly increased.

As a result, the combination of random data extraction, an appropriate algorithm model, and the sliding window mechanism will make great changes to the forecast problem of the compressor geometric variable system. Moreover, this system can serve as a reference for other systems to use this forecasting method for making similar predictions or other research activities.

### 3.2 The enhanced ConvNeXt network

**Fig 4:**
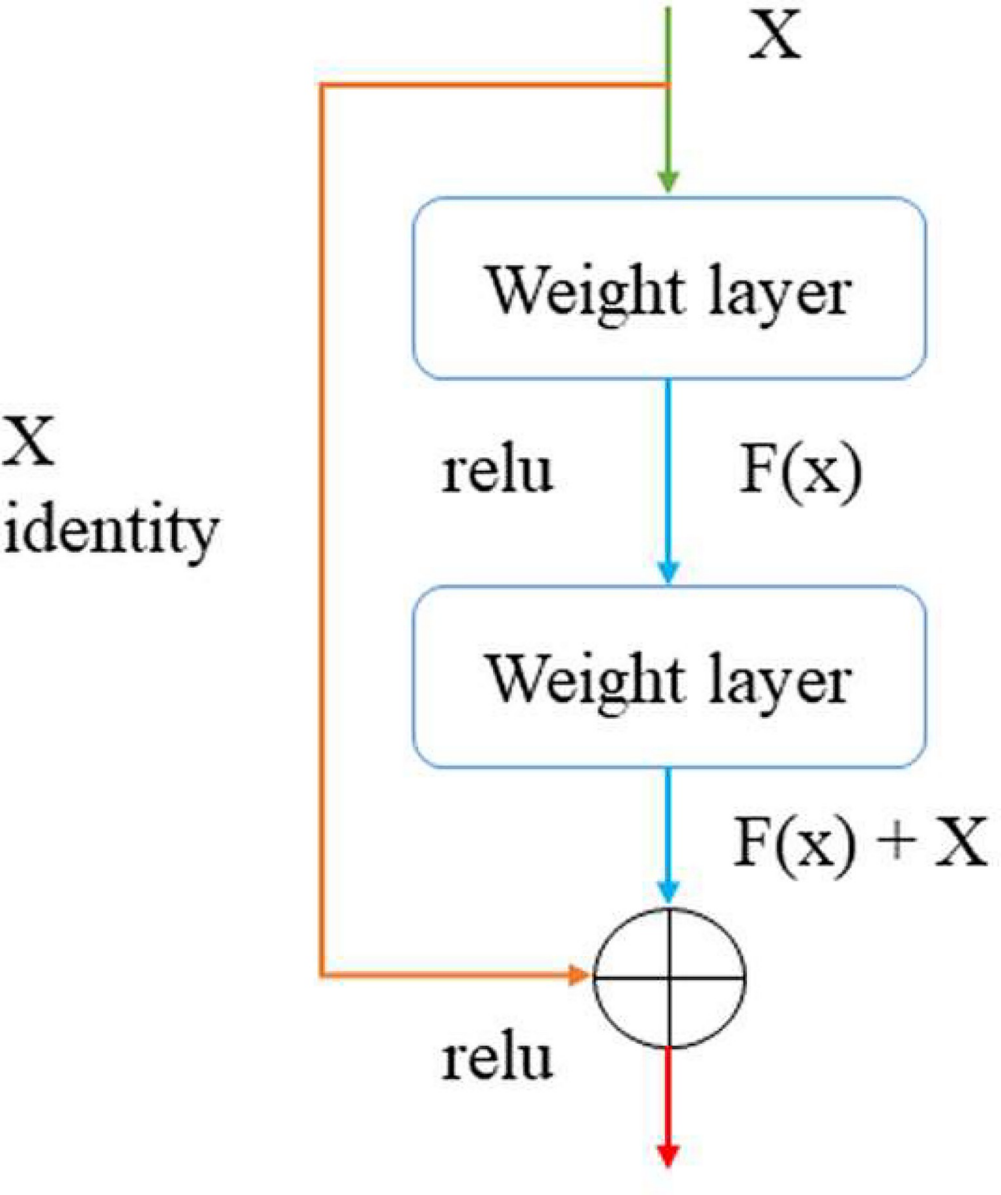
Residual learning: A building block.

After He [17] et al proposed the classic neural network, Residual Network (ResNet), this architecture has had a great impact on the computer vision (CV) field and continues to this day. The structure of this model is shown in Fig 4. This network learns the residual F (x) between H (x) and X, not directly obtaining the expectation function. By learning the residual to reach the optimal function step by step, the model with the smallest error can be obtained.

**Fig 5:**
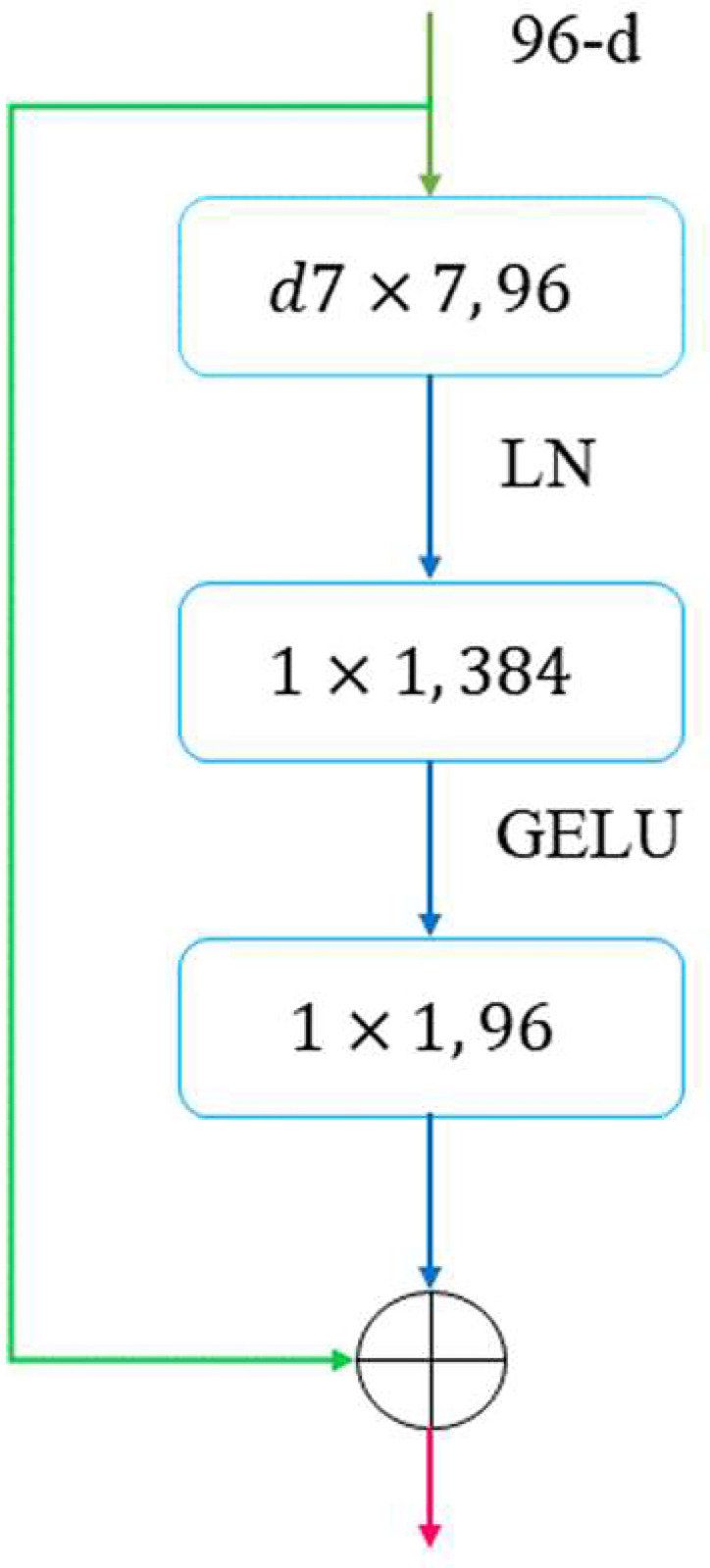
The enhanced residual block.

Under the tide of Transformer sweeping the field of deep learning, the ConvNeXt model [18] which is based on a ResNet proposed by FAIR has achieved the same effect as the Swin Transformer [19]. This finding shows that the ResNet still has a large potential. Based on the residual idea, it improves the model’s structure, makes the overall structure more excellent and reasonable, and updates and modernizes associated components. And since the ViT model introduced the Transformer structure into CV, the following MAE model and Swin Transformer model are derived and developed on this basis. The most important step of the ViT model is to divide the image into patches, that is, different regions, and process them in patches [20]. The traditional ResNet model uses a 7x7 convolutional layer maximum pooling layer to directly process the image as a whole. And the ConvNeXt model adopts this kind of idea. It first processes the input with a 4x4 convolutional layer and processes the information of one patch at a time. Therefore, it effectively reduces the down sampling times and improves the model performance. Additionally, the network width is improved by increasing the number of channels from 64 to 96. The processing of one channel by each convolutional kernel is independent of the other channels, just like the self-attention mechanism. One channel contains mixed weighted spatial information. In addition, the residual module is changed as shown in Fig 5, so that information loss caused by compressed dimensions can be avoided when converting information between feature spaces of different dimensions. Finally, some small modules are modernized, such as replacing ReLU with GELU and reducing the number of active layers.

**Fig 6:**
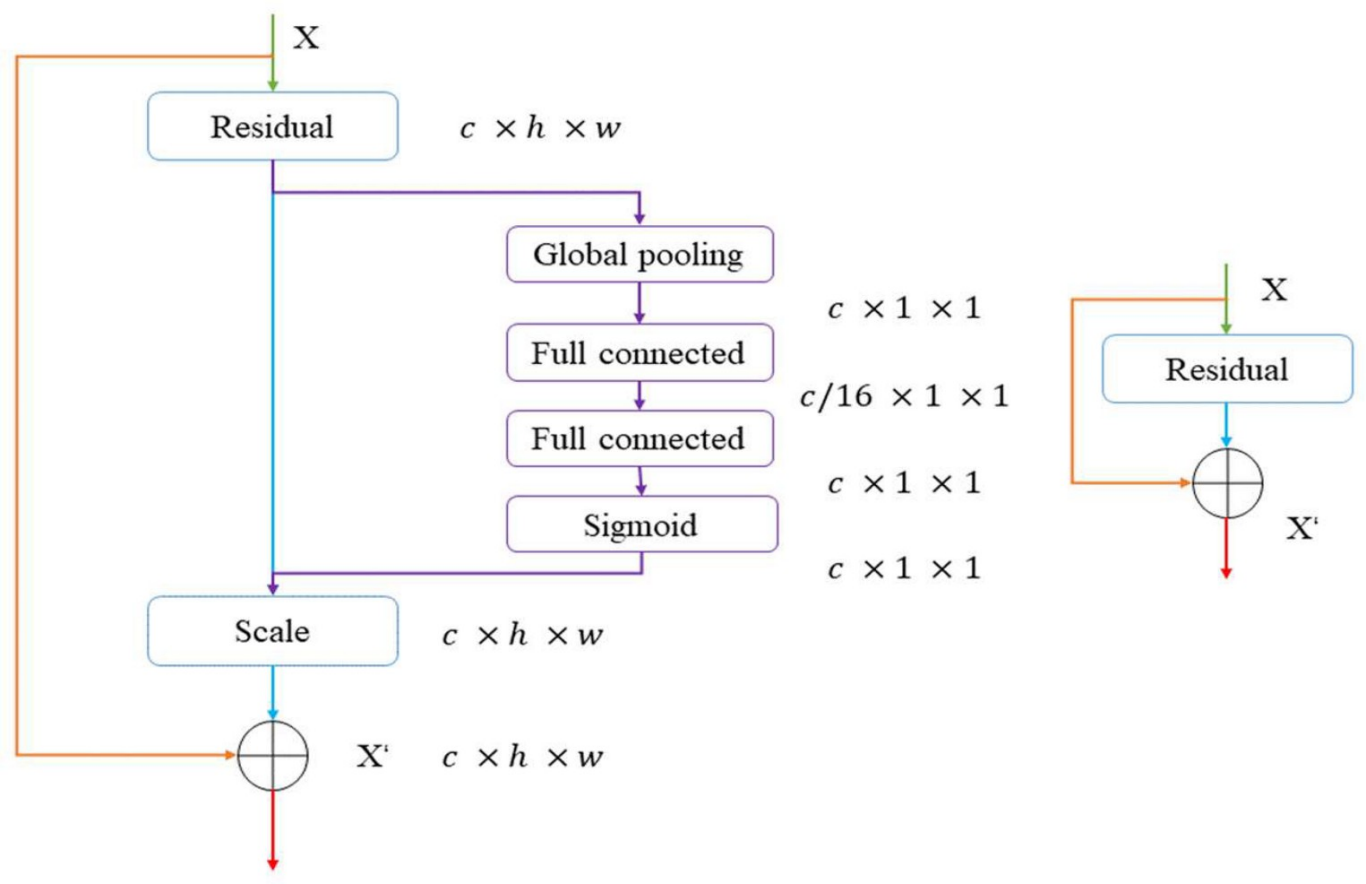
SE-ResNet module.

To improve the performance of the ConvNeXt model. A SE (Squeeze-and-excitation) block [21] which was the champion of the last ImageNet 2017 competition will be added to the ConvNeXt model. Fig 6 shows its structure. This global architecture based on a residual block ensures the model’s excellent performance. Through Squeeze and Excitation techniques (See Formula 1 and Formula 2), these two ideas explicitly model channel interdependencies within modules.


$$z_{c}=F_{sq}(u_{c})=\frac{1}{H\times W}\sum_{i=1}^{H}\sum_{j=1}^{W}u_{c}(i,j),z\in R^{C}$$ (1)

s=Fex(z,W)=σ(W2ReLU(W1z)) (2)

$$W_{1}\in R^{\frac{C}{r}\times C},W_{2}\in R^{C\times \frac{C}{r}}$$

Furthermore, a strategy called “Feature recalibration” looks like an attention mechanism in a sense, which means the block automatically acquires the importance of each feature channel via learning, and then improves the useful features according to this importance and suppresses the features that are not useful for the current task. To meet our research goal, we have slightly modified these models to better match our study.

### 3.3 Optimizer and learning rate control strategy

**Fig 7:**
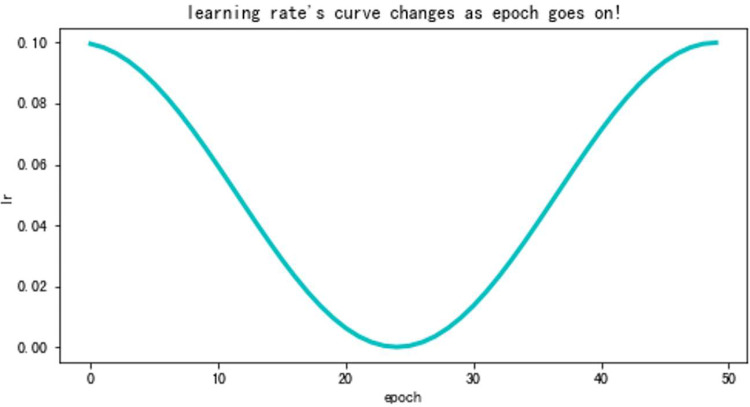
Cosine Annealing LR (T max = 25).

The optimizer we used in this study is Adaptive Moment Estimation with decoupled weight decay (AdamW) [22]. Hyperparameters *β*_1_, *β*_2_ and Weight Decay are set as 0.9, 0.999 and 0.05 for all tasks. And to better train our model, we adopt Cosine Annealing LR to control our learning rate. And according to Formula 3, we could show the variation in general trends of learning rate when we are training models (see Fig 7). The meaning of these parameters will be presented in Table 3. And hyperparameter T max is equal to training epochs.


$$\eta_{lr}=\eta_{\min}^{i}+0.5\times (\eta_{\max}^{i}-\eta_{\min}^{i})\times (1+(\cos\frac{T_{cur}}{T_{i}}\pi ))$$ (3)

**Table 3:** Parameters of Cosine Annealing LR.

Parameter	meaning
$\eta_{min}^{i}$	Minimum learning rate
$\eta_{max}^{i}$	Maximum learning rate
*T*_*cur*_	Current epoch
*T*_*i*_	Number of periods

## 4. Method validation

In our experiments section, the feasibility of high accuracy forecast must be tested by mainly comparing our method with the traditional method, which could present how accurate our method is. It should be noted that in order to explore the possibility of forecasting the compressor geometric variable system, the input and output steps are set at 100 and 10 respectively. With this setting, the information of the input data might be adequate to make the model more accurate. Furthermore, experiments about different aero-engine types and those two aero-engine states will also be discussed. By doing this, the method’s applicability and reliability will be significantly strengthened. It can also demonstrate the learning algorithm’s potent potential, showing that both steady state and transition state features can be learned concurrently.

It should be noted that the datasets we used in this paper are neither standardized nor normalized, the angle (°) of VSV and the opening (°) of VBV can be directly reflected in the output. And the Mean Square Error (MSE) will be used to measure the performance of our results. Hence, MSE is the square of the angle (°) and the opening (°). Finally, the X-axis step in the following figure represents the change over time.


$$\frac{1}{m}\sum_{i=1}^{m}{\left(y_{i}-{\hat{y}}_{i}\right)}^{2}$$ (4)

Meanwhile, all MSEs are yielded by the mean error of 100 different offset experiments which use the minimum test error model. And the different offsets will make the data input to be different. But all input data comes from the same test datasets and has only the offset. Therefore, the offset will make the extracted data different every time but retain the same test data source. It should be noted that the unit of VSV and VBV systems predicted in this paper is the degree (°), so the Y-axis of the trend chart is this unit. And the gray area between different curves is the difference between the true and the forecasted.

### 4.1 Feasibility verification

In this paper, verifying the feasibility of our idea is the first thing needed to do. In this part, not only is the feasibility of the novel method tested but it is also compared with the errors of the traditional method. Under the same conditions which are all models trained completely but not overfitting, the average value of 100 tests with the offset will be used as a measure of the performance of each model. This might make results as accurate as possible. Table 4 presents the results of different types of aero-engine VSV and VBV forecasting experiments using the enhanced ConvNeXt model. And Table 5 shows the comparison result on the compressor geometric variable system of the Type A aero-engine.

**Table 4:** Comparison results (MSE) of the two method.

Type	System	Loss (Our)	Loss (Traditional)	Percentage (Our / Tra)
A	VSV	0.527	2.209	23.85%
A	VBV	2.381	7.131	33.40%
B	VSV	0.892	4.447	20.07%
B	VBV	108.837	136.930	79.48%

**Table 5:** Comparison results (MSE) of different models.

Type	System	Model	Loss (MSE)
A	VSV	Enhanced ConvNeXt18	0.526652
A	VSV	ConvNeXt18	0.604219
A	VSV	SENet18	0.501077
A	VSV	ResNet18	0.488490
A	VBV	Enhanced ConvNeXt18	2.381444
A	VBV	ConvNeXt18	2.579979
A	VBV	SENet18	2.003873
A	VBV	ResNet18	2.3260968

**Fig 8:**
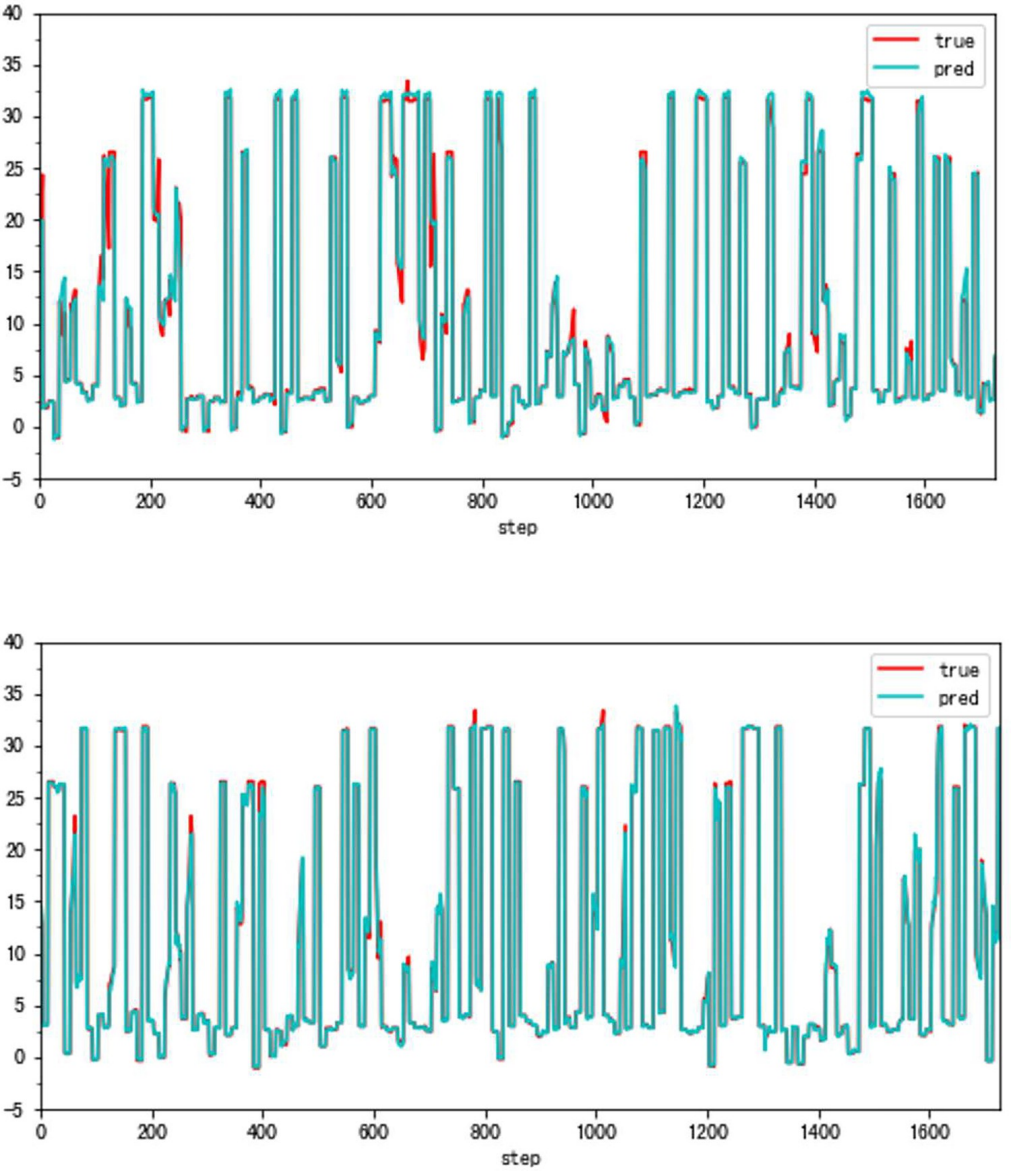
a. The result of the traditional method (VSV, Type A). b. The result of the high accuracy method (VSV, Type A).

**Fig 9:**
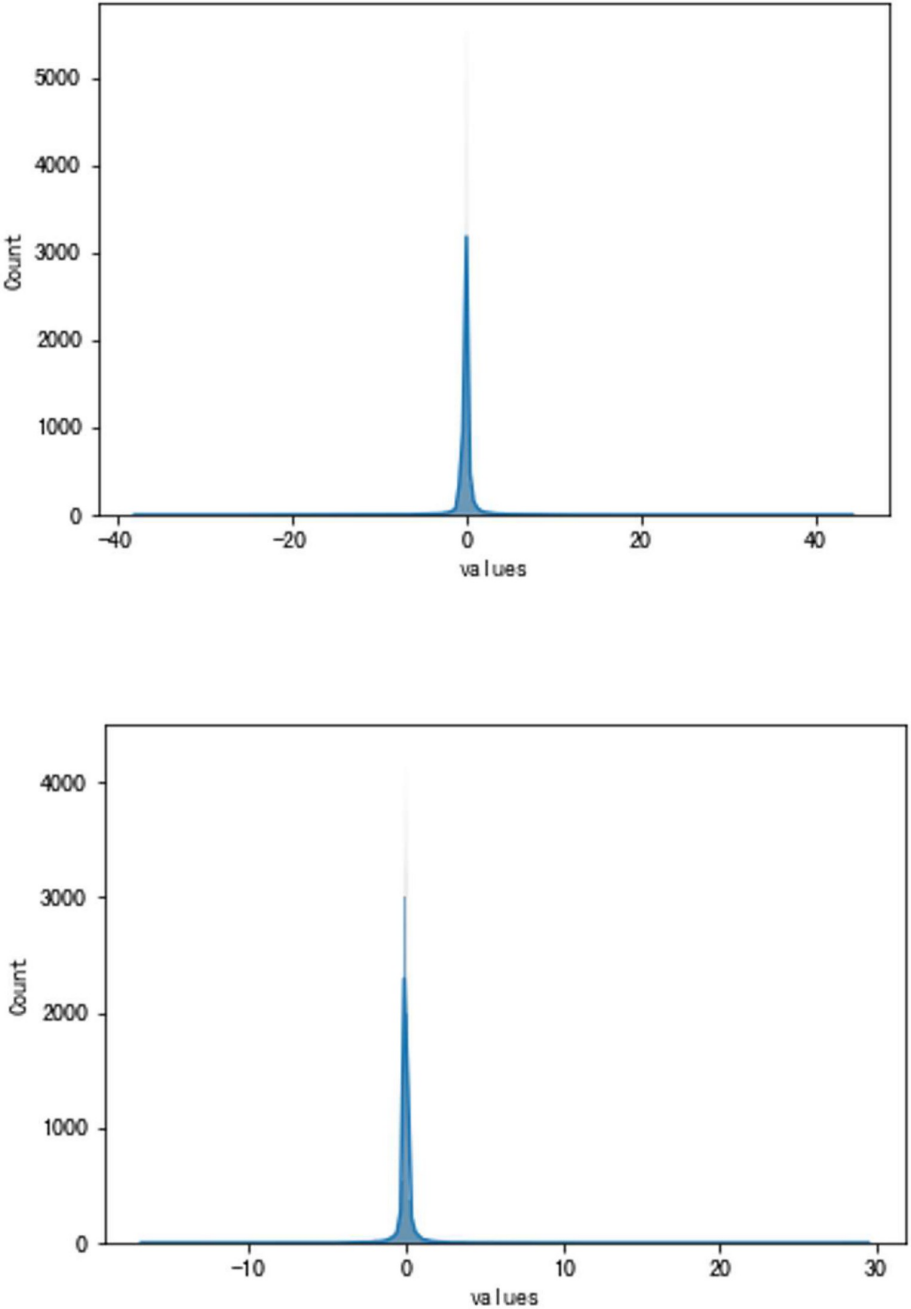
a. The distribution of traditional method errors (VSV, Type A). b. The distribution of high accuracy method errors (VSV, Type A).

**Fig 10:**
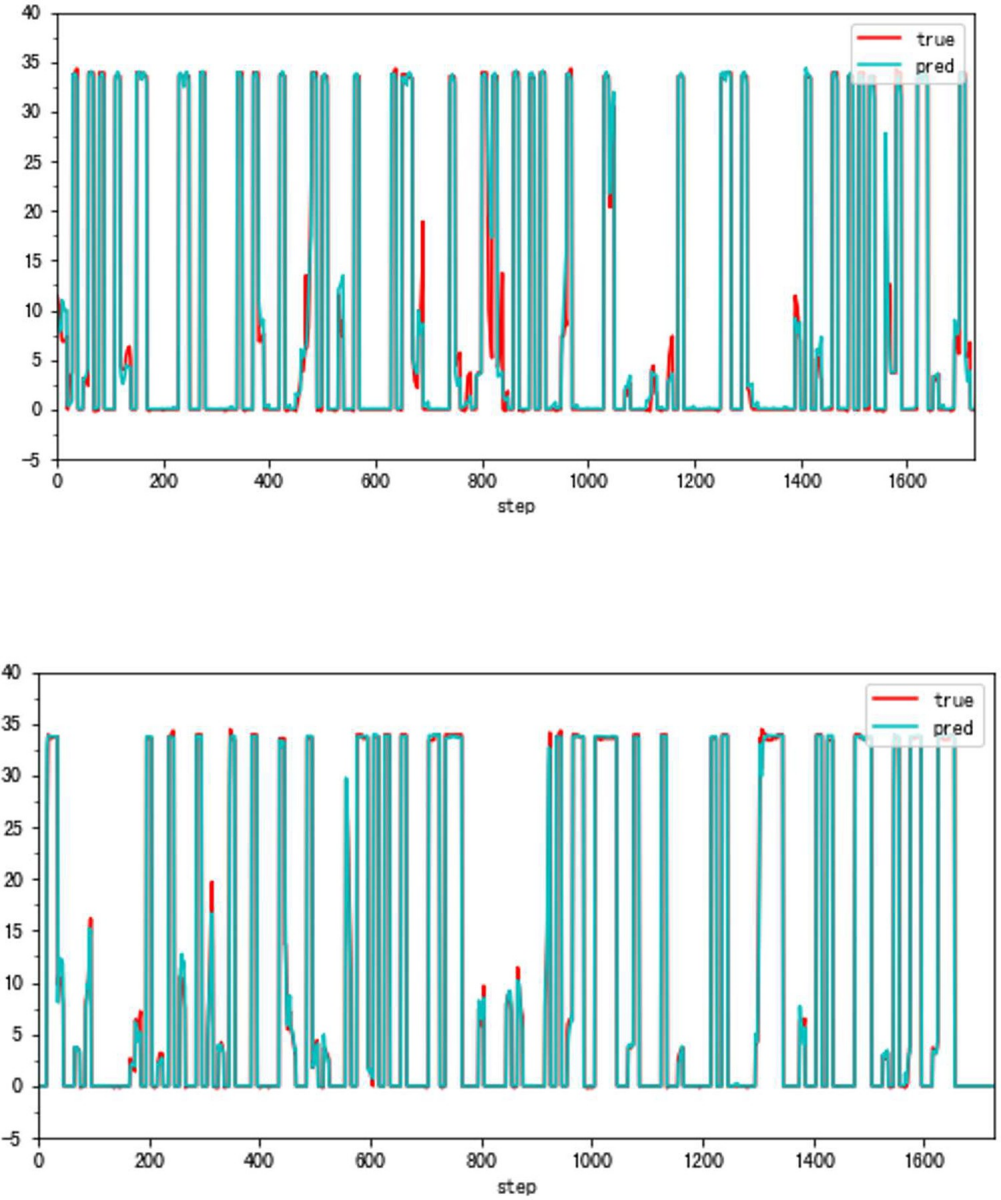
a. The result of the traditional method (VBV, Type A). b. The result of the high accuracy method (VBV, Type A).

**Fig 11:**
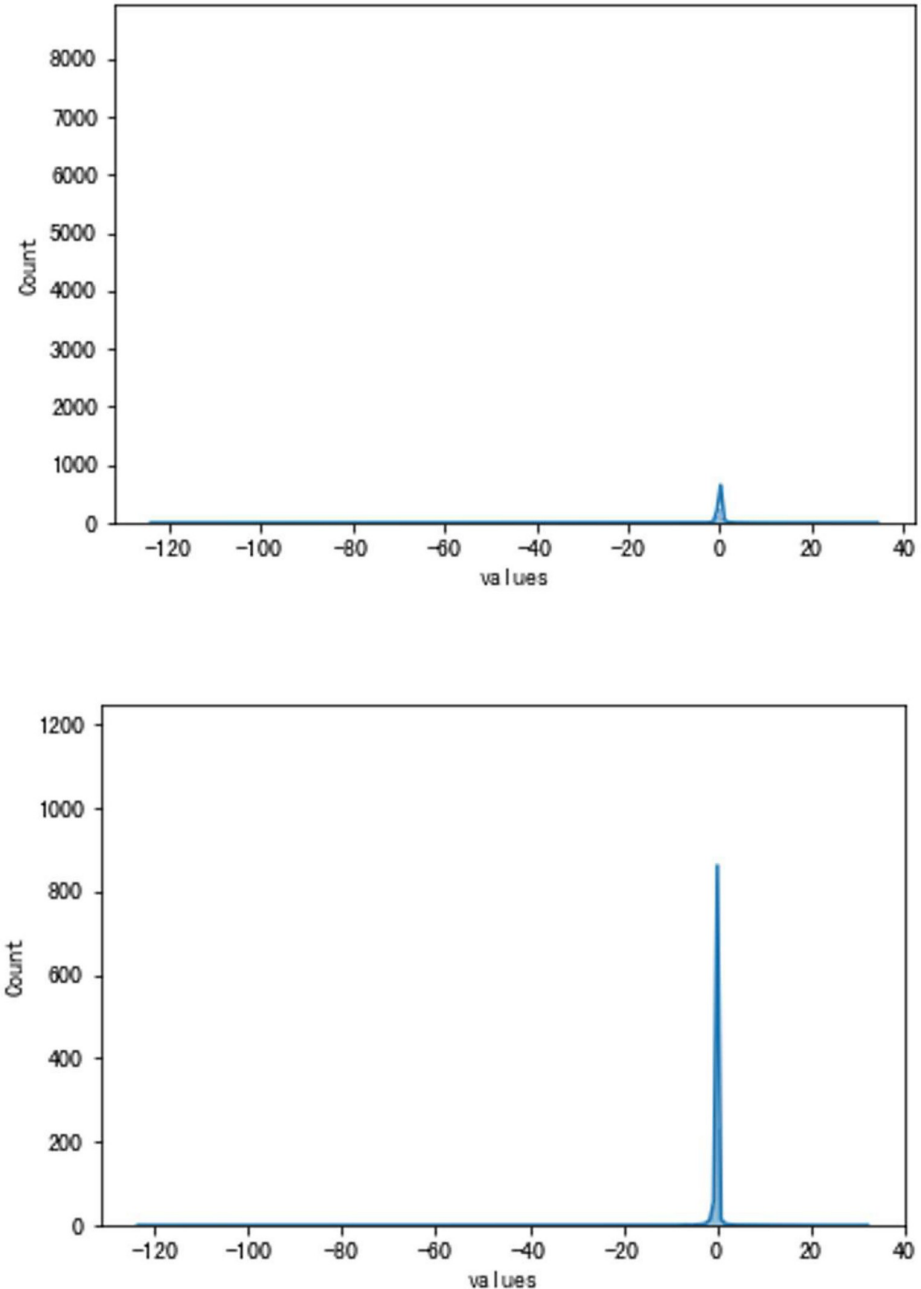
a. The distribution of traditional method errors (VSV, Type A). b. The distribution of high accuracy method errors (VBV, Type A).

As can be seen, the error of the enhanced ConvNeXt model with the sliding window mechanism is indeed far less than that of the direct forecasting method. The new method is about 23.85% of the traditional in the experiment of forecasting Type A aero-engine VSV angles and around 33.40% of the traditional in Type A VBV experiments. Additionally, our model has shown superior outcomes when both use the sliding window mechanism. Figs 8–11 present results of forecasting the compressor geometric variable system on the Type A aero-engine.

It can be seen from the results in this part that the forecast of Type A aero-engine s VSV angles and VBV openings are greatly accurate. Essential features of the forecast have been fully learned by the enhanced ConvNeXt model with the sliding window mechanism. As a result, the accuracy of these forecasts may reach an extremely high level. Based on the results, the forecast of the future trend of the change of VSV angles and VBV openings is greatly accurate, and tracking the sudden change is timely enough.

It is obvious that this accomplishment cannot be achieved without future information from other factors. This might prompt people to make some limited predictions on the early warning of the compressor geometric variable system to avoid insecure situations. The deviation between forecasting values and true sensor signal values could be measured in real-time. If the deviation within one step or accumulated over several steps is unacceptable, the early warning system can alert the crew. In this way, the crew might manage the hazardous situation in time.

### 4.2 Applicability verification

**Fig 12:**
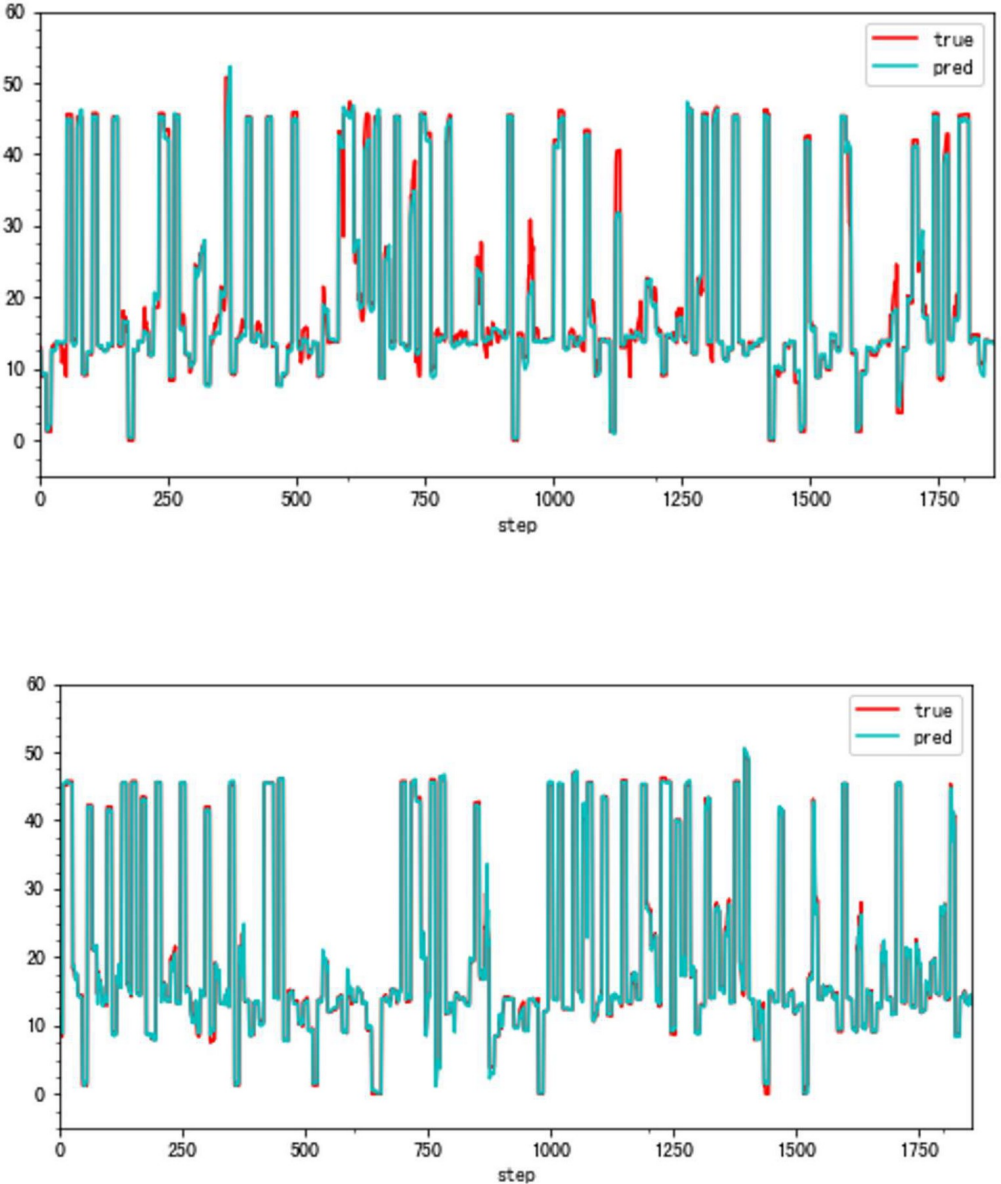
a. The result of the traditional method (VSV, Type B). b. The result of the high accuracy method (VSV, Type B).

**Fig 13:**
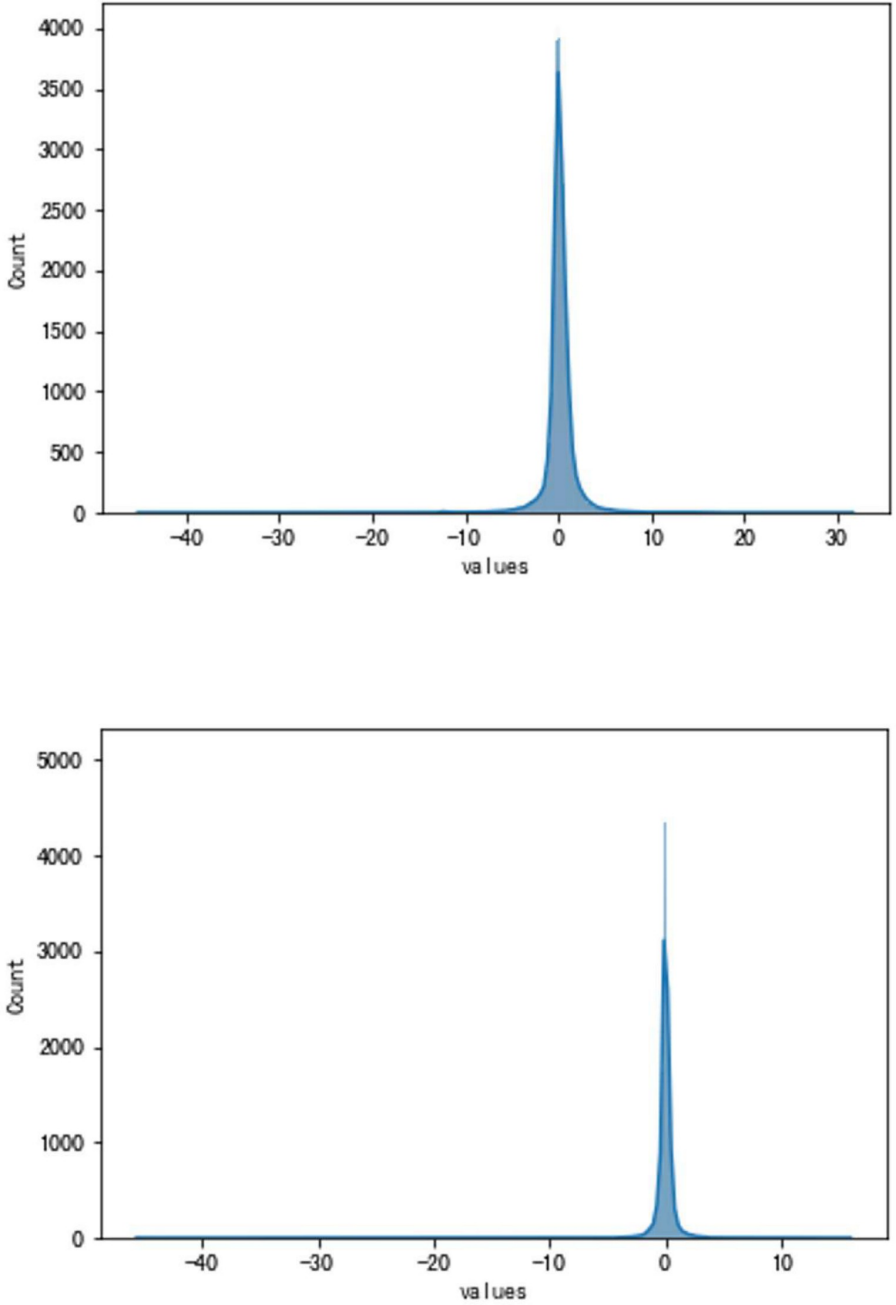
a. The distribution of traditional method errors (VSV, Type B). b. The distribution of high accuracy method errors (VSV, Type B).

**Fig 14:**
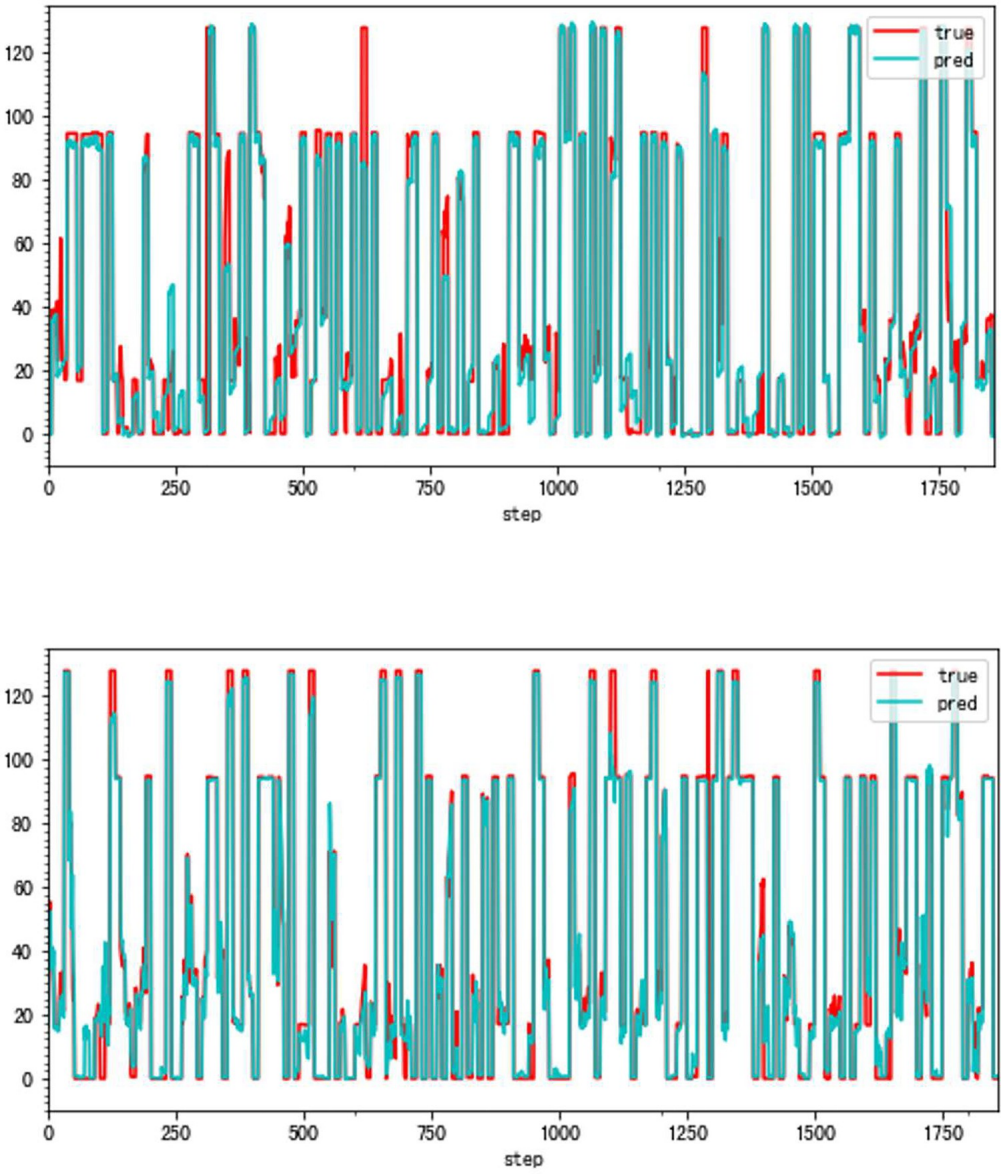
a. The result of the traditional method (VBV, Type B). b. The result of the high accuracy method (VBV, Type B).

**Fig 15:**
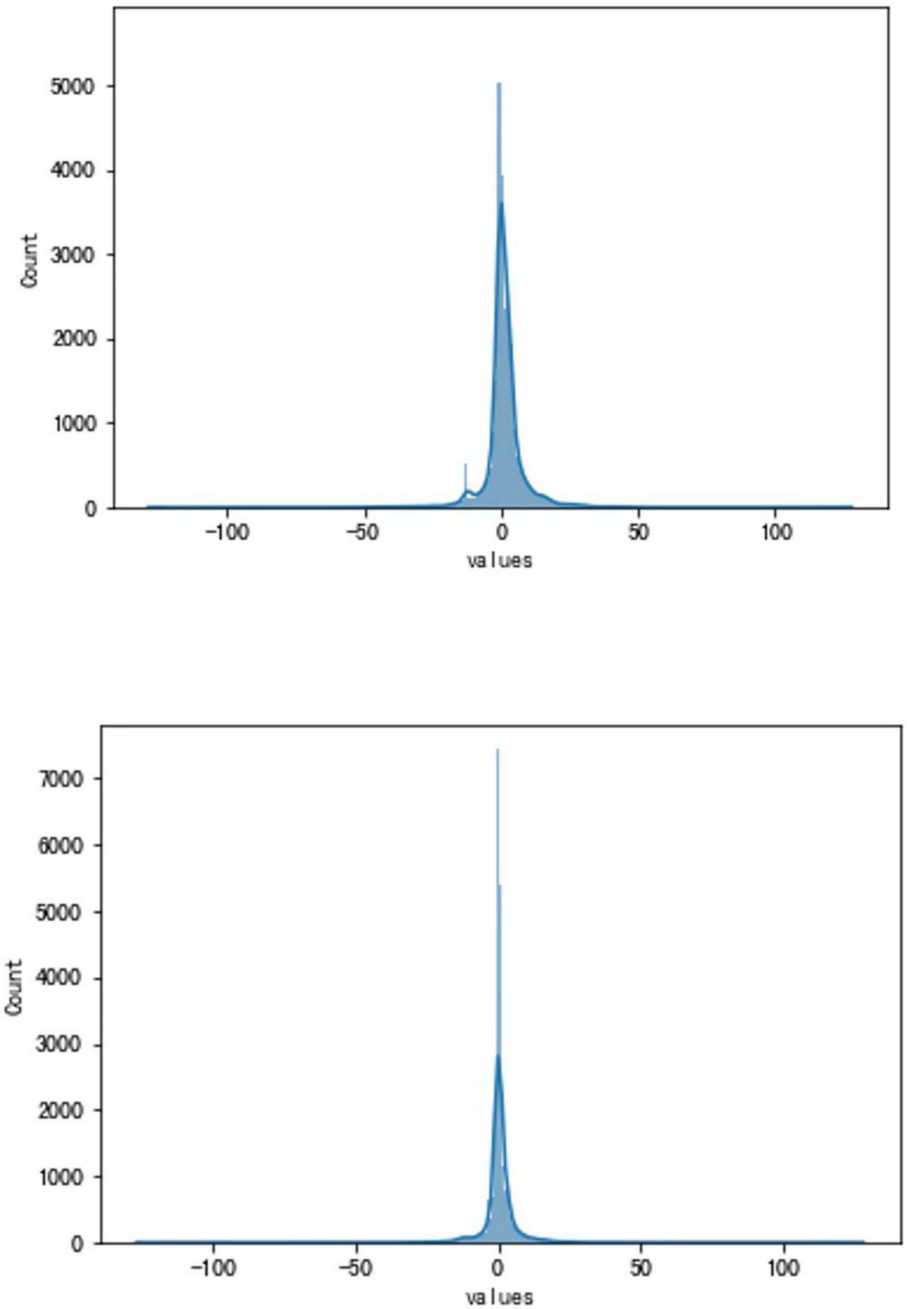
a. The distribution of traditional method errors (VBV, Type B). b. The distribution of high accuracy method errors (VBV, Type B).

Although the very accurate method may produce excellent results in the sort of aero-engine mentioned above, its application to other types of aero-engines needs to be verified. To this end, the same experiments are carried out on a Type B aero-engine and tried to make sure that the experimental conditions were similar. Table 4 presents the numerical improvement of forecasting accuracy. The forecasting errors of VSV angles and VBV openings are 20.07% and 79.48% of those traditional methods, respectively. To observe this result more intuitively, Figs 12 and 13 (as same as Fig 1) show the changes in the angle of VSV. And Figs 14 and 15 present the opening of VBV resulting from these two completely different approaches.

According to these results, it is obvious that the combination of the sliding window mechanism and the enhanced ConvNeXt model can truly make a great leap in the problem of improving forecast accuracy. The idea of adding other parameters’ information while forecasting significantly reduces predictive errors. However, it is should be noted that the forecast of the VBV opening of Type B aero-engines has a relatively big error. There might be two reasons for this phenomenon. First, the VBV opening has a wide range of changes (0° to 135°), and the result is that the square of the degree can lead to an intuitively large numerical value. Second, some essential parameters may be omitted, which means that the model does not fully learn the changing features of the VBV opening. But the parameter selection is not the key point in this article and it is a big story, hence this requires a lot of research in the future. Third, the reason why a light model (ConvNeXt18) is chosen is the potential for insufficient data. However, the light model might also run the risk of not being complex enough to fully learn the vital features. Meanwhile, there is a risk of an inadequate amount of data. After all, millions of data just reach the threshold of the modern deep learning area. Comparing Part 4.1 and this part, results show that the parameter selection and the data volume might be latent reasons.

Although there may be various reasons above, our novel method still shows a strong capacity for forecasting aspects and greatly improves forecasting accuracy. As a result, the future information introduction of other parameters is extremely useful.

This is an interesting finding as the errors are surprisingly small. Consequently, this highly accurate method might have wide applicability. And the reason why the error of the Type A aero-engine is much smaller than that of Type B is probably that the parameters we select could be more suitable for Type A aero-engines. The parameters of different types of aero-engines are a little different, and the interaction between them is also a very profound knowledge. It is also a problem whether the selection of parameters in this paper is comprehensive. After all, this paper only selects ten parameters to explore our goal, and the aero-engine is a huge and complex system. Although end-to-end learning of deep learning can eliminate part works of feature engineering to a certain extent. However, to thoroughly study this problem, professional aeronautical knowledge and multi-field research background are necessary.

Most significantly, various aero-engine types have various control systems. The main cause of the parameter selection issue is this. But this provides future research an inspiration, which could be applied in the design of control systems to optimize the control process.

Through Part 4.1 and Part 4.2, how important it is to add the future information of other parameters to the forecasting process can be seen. With the feedback of the sliding window mechanism, the model can correct information errors while forecasting. This strategy allows the model to perceive sudden changes in the future time span (like states change) that require to be forecasted. As a result, an enhanced ConvNeXt model with the sliding window mechanism, adding the future information of other parameters, is a greatly useful and highly accurate method.

### 4.3 Aero-engine states analysis

In this Part, to make our thoughts more reasonable and convincing, two groups of state experiments on VSV and VBV are shown later based on the Type A and the Type B aero-engine. It should be noted that the triangle in all box plots represents the position of the average value. Circles represent outliers.

**Fig 16:**
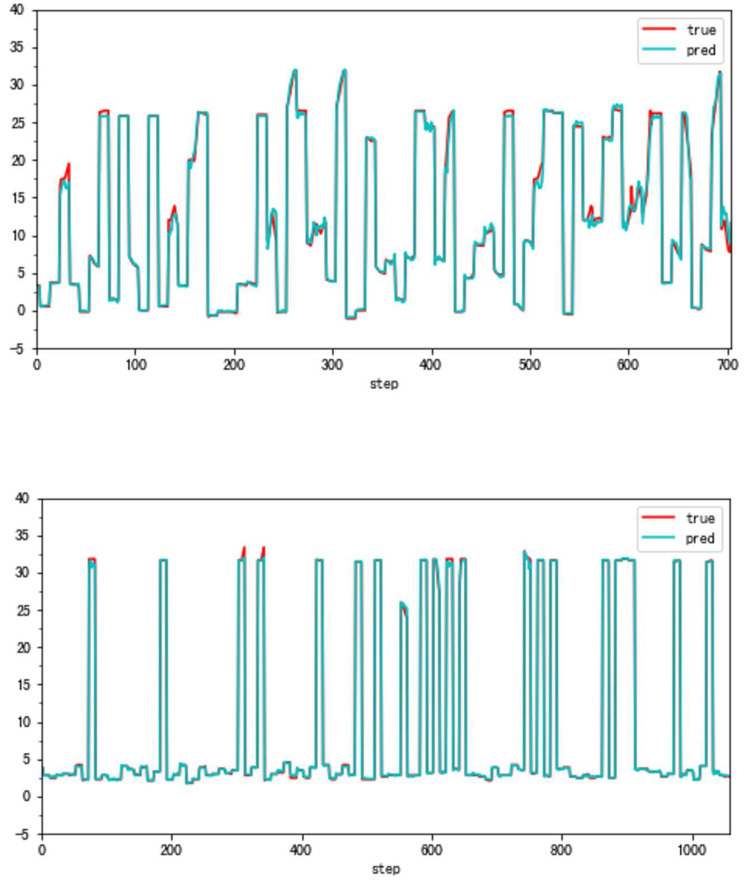
a. The transition state (VSV, Type A aero-engine). b. The steady state (VSV, Type A aero-engine).

**Fig 17:**
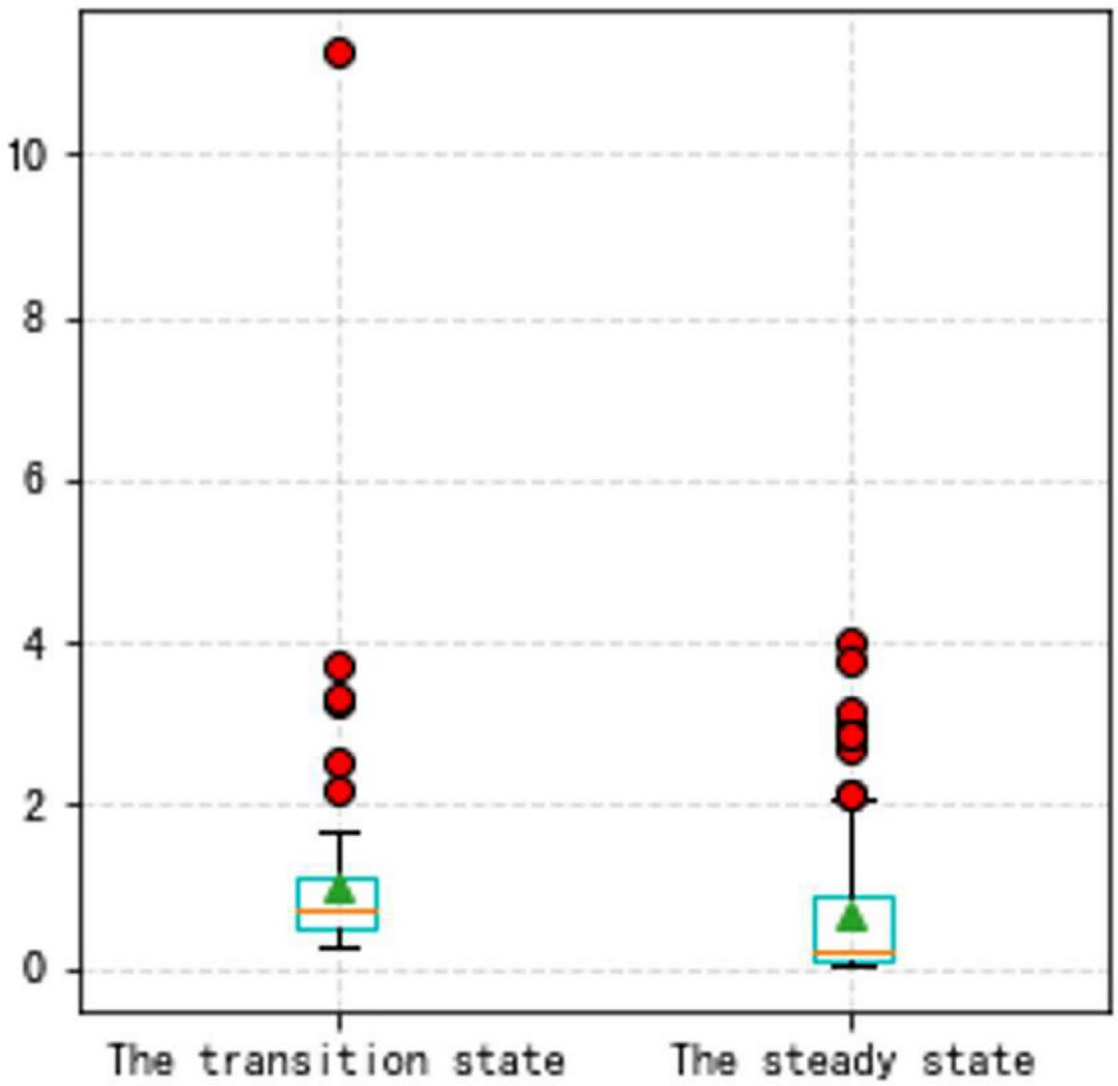
The box plot of the two states (VSV, Type A aero-engine).

**Fig 18:**
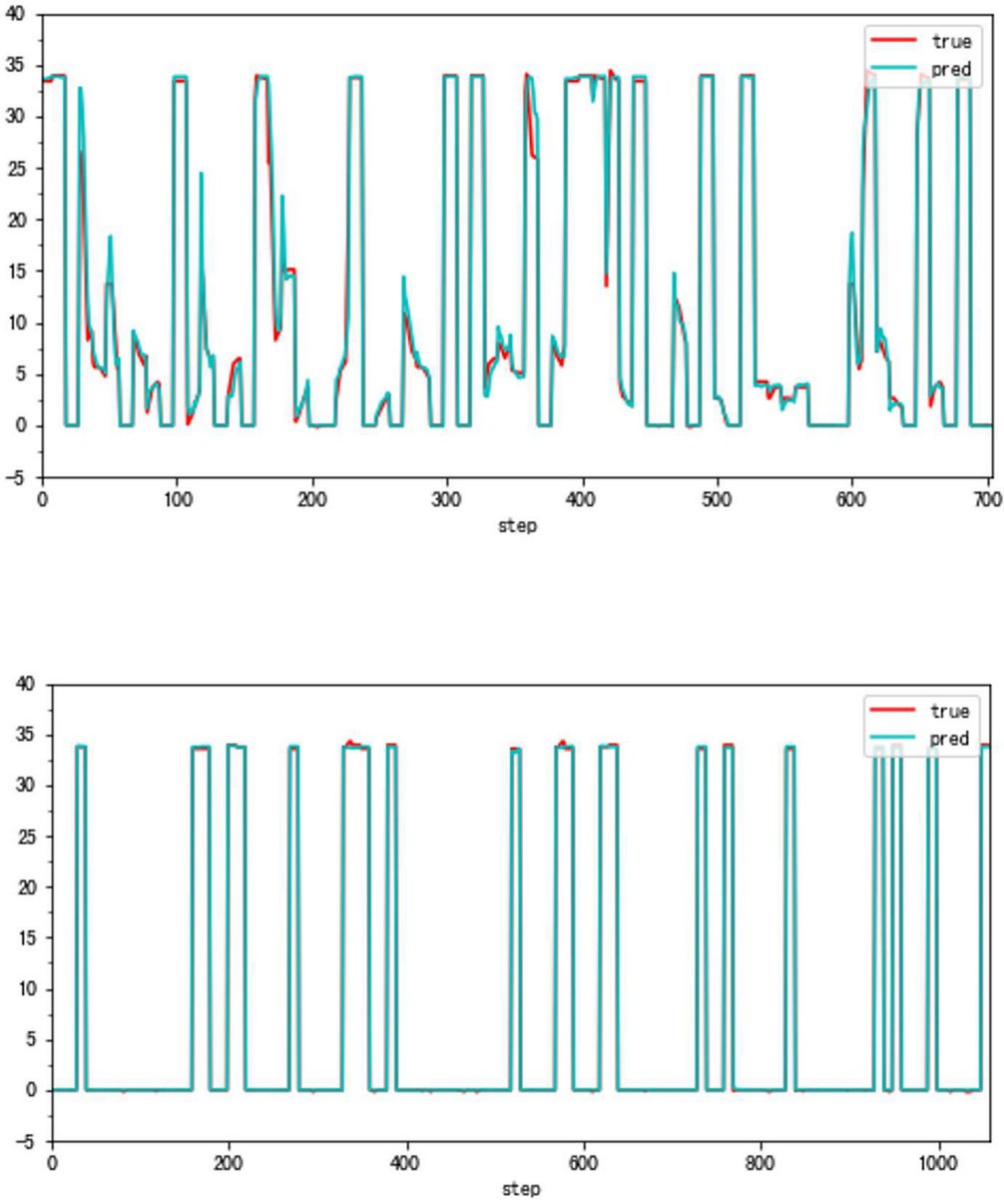
a. The transition state (VBV, Type A aero-engine). b. The steady state (VBV, Type A aero-engine).

**Fig 19:**
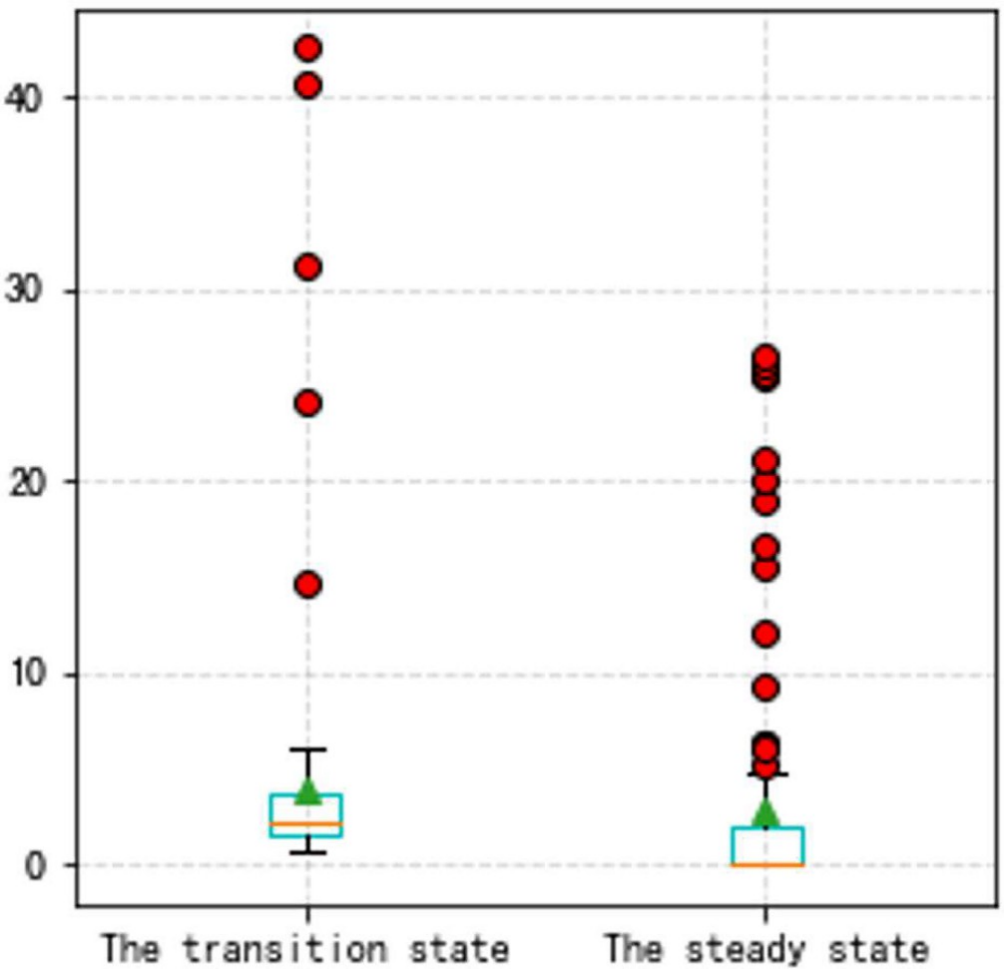
The box plot of the two states (VBV, Type A aero-engine).

**Fig 20:**
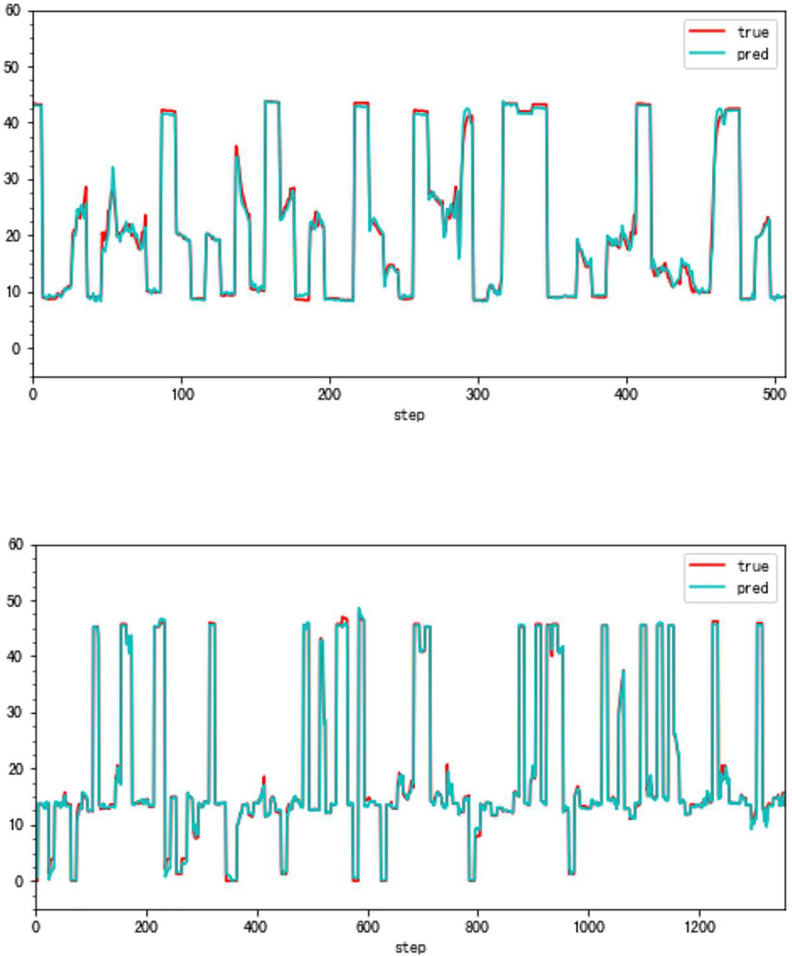
a. The transition state (VSV, Type B aero-engine). b. The steady state (VSV, Type B aero-engine).

**Fig 21:**
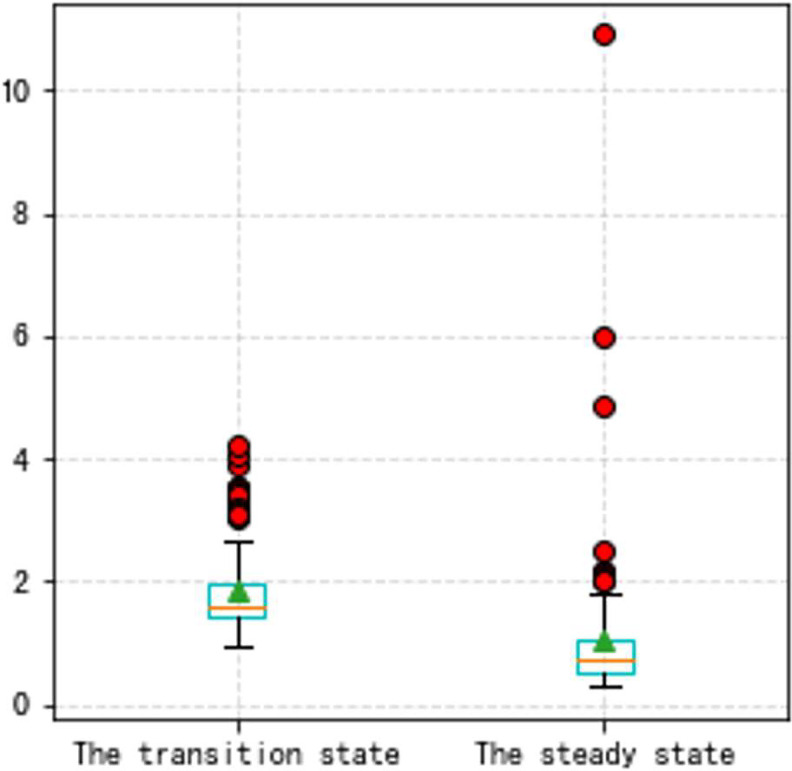
The box plot of the two states (VSV, Type B aero-engine).

**Fig 22:**
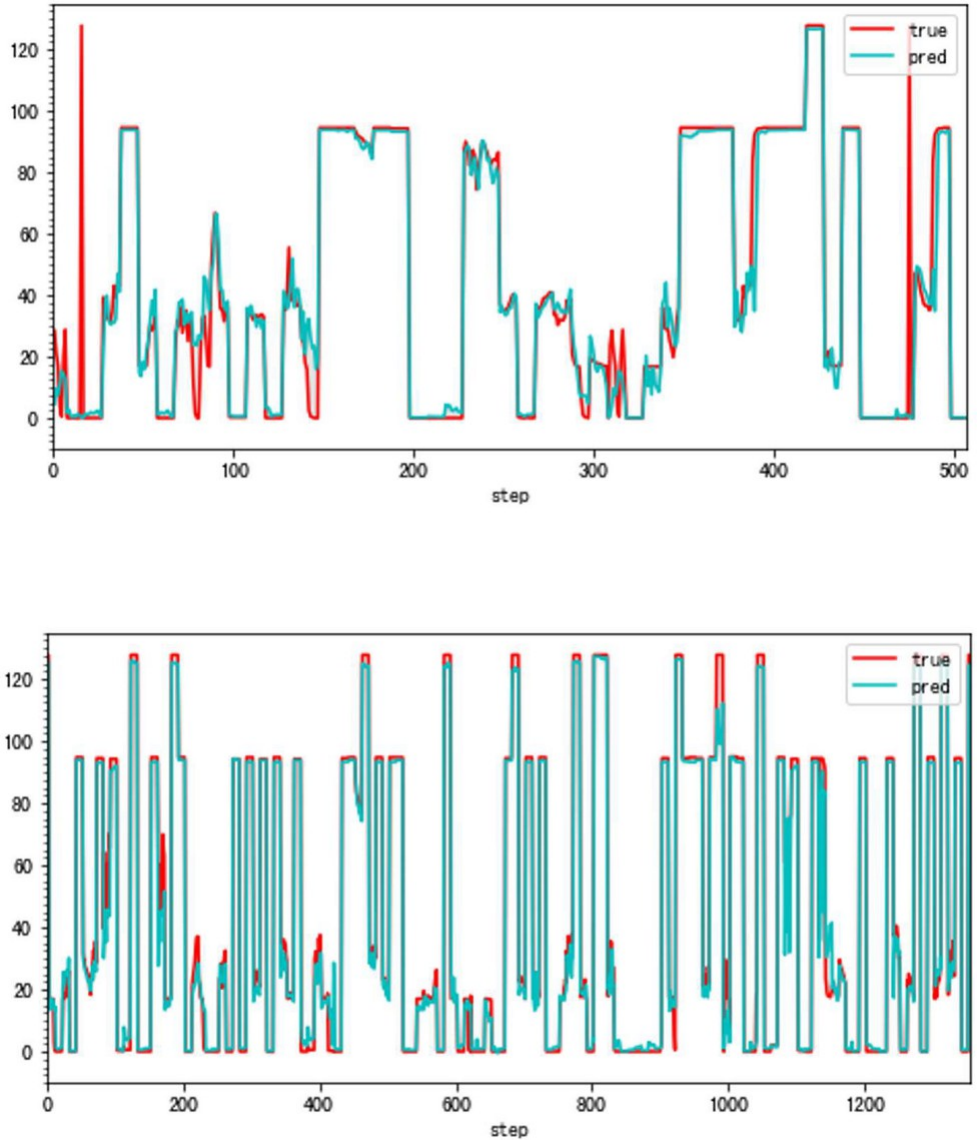
a. The transition state (VBV, Type B aero-engine). b. The steady state (VBV, Type B aero-engine).

**Fig 23:**
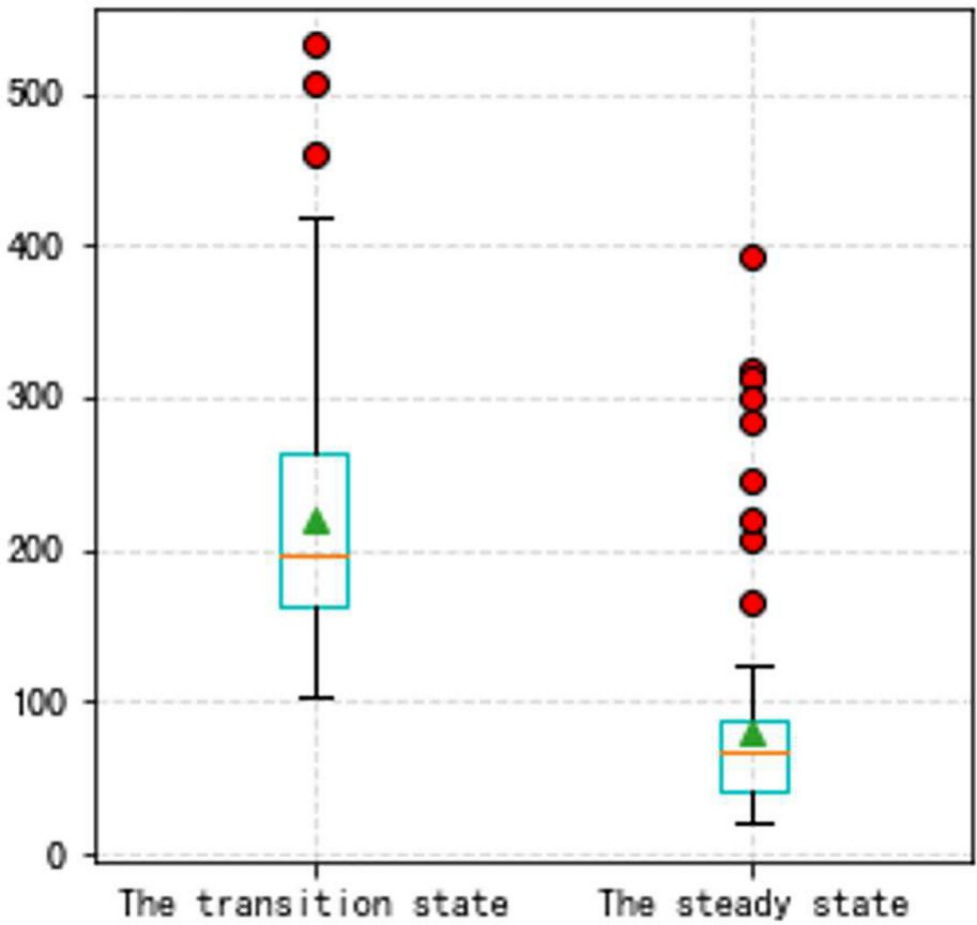
The box plot of the two states (VBV, Type B aero-engine).

From Figs 16–23, it can be seen that regardless of whether the transition state prediction and the steady state prediction, the VSV angle or the VBV opening can be forecasted accurately. And the forecasting errors of the steady state is lower than the transition state. But there is a relatively counterintuitive phenomenon here. When the two states are forecasted separately, the predictive errors are greater than the initial entire random sampling errors. This might be brought on by randomly extracting data, although it seldom affects the outcomes. The accuracy of the forecast is fairly high, and the model can track sudden changes immediately in the transition state.

As a result, Experiments show that the model can learn the features of these two states well, and can make high-precision predictions according to the input data. Hence, the influence of aero-engine states on forecast accuracy can be set aside for the time being. And the model trained by our method can be regarded as a general state model. It is extremely useful in real industries.

## 5. Conclusion

Aiming at forecasting the compressor geometric variable system more accurately, this article proposed a novel method by using an enhanced ConvNeXt model that utilizes the Sliding Window Algorithm mechanism. The results demonstrated that it has been successfully used to forecast the compressor geometric variable system in various types of aero-engine on a high accuracy level. And different types of real aero-engines are used in the experiment, which is uncommon. Additionally, the aero-engine has specific criteria for its own condition in the real world. The aero-engine reacts to various circumstances in various states. Both transient and steady states are included in the aero-engine state. This paper, like previous relevant experiments, did not separate the aero-engine state in the feasibility and applicability tests but instead established a general model by randomly extracting a block of data. But carrying out experiments additionally for various aero-engine states to demonstrate the validity of this idea.

This article has shown that ①It is possible to accurately forecast the compressor geometric variable system’s future trend. And a highly accurate method for this system is proposed in this paper based on an enhanced ConvNeXt model that makes use of the sliding window mechanism. According to the results, when compared with the traditional prediction method, the loss (MSE) of our novel predictive method is only **20.07%** (VSV, Type B aero-engine) of that of the traditional under the same conditions. ② This approach can be used for various sorts of aero-engines (Type A), and it might also produce quite impressive results (VSV: 0.527, **23.85%** and VBV: 2.381, **33.40%**). And that is the applicability how to be proved. This concept can therefore be applied to relevant studies in the realm of aviation. It might give researchers insight into how to study various systems. ③This article also tests the model’s ability to recognize various aero-engine operating states and to generate precise forecasts. Therefore, there are adequate grounds to believe that when there is an abundance of data, a sufficiently complicated algorithm model can simultaneously learn the features of different states.

The findings presented here provide new insight into the compressor geometric variable system’s early warning mechanism for avoiding risky circumstances. The difference between signal levels and forecasts could be timely monitored. The early warning system can alert the crew if the deviation within one step or aggregated over numerous steps is unacceptable. This would enable the staff to address the hazardous issue immediately. Moreover, this highly accurate method could expand its application scope and be applied to other systems for research. Additionally, this extremely accurate technique can broaden the breadth of its study applications. The engine design process will undoubtedly be fed back into, assisted by, and raised in quality when this very accurate forecasting method is integrated with fault diagnosis, fault prediction, fault classification, and other technologies. As a result, this new method could play an auxiliary role in the aero-engines design process.

In addition to the above, this paper still has a few issues that need to be resolved. One of the limitations of this study is the data scale used in the paper, which is a million that has initially reached the scale of deep learning. If more datasets can be gathered, the outcome of the forecast could be more accurate. Second, the mechanical error cannot be completely avoided, and this could have an impact on our research. Moreover, choosing the right parameters is a major challenge that requires in-depth research. In this work, we made an effort to choose the appropriate parameters based on previous research, the aero-engine design concept, and expert manuals. There are still some shortcomings and irrationalities even if it is not the main topic of this essay (the main goal is to offer a highly accurate method for the forecast).

Notwithstanding these limitations, the study suggests that this would be a fruitful area for further work. In follow-up research, how to apply this method to other essential systems will be mainly concerned. And the hidden relationship between time span and control systems will be given a lot of thought.

## Supporting information

S1 File (ZIP)
